# Mitochondrial Dysfunction is a Key Pathway that Links Saturated Fat Intake to the Development and Progression of NAFLD

**DOI:** 10.1002/mnfr.201900942

**Published:** 2020-07-13

**Authors:** Ruth C. R. Meex, Ellen E. Blaak

**Affiliations:** ^1^ Department of Human Biology, NUTRIM School of Nutrition and Translational Research in Metabolism Maastricht University Universiteitssingel 50 Maastricht 6229 ER The Netherlands

**Keywords:** human intervention studies, mitochondrial dysfunction, non‐alcoholic fatty liver disease, nutrition, saturated fatty acids

## Abstract

Non‐Alcoholic fatty liver disease (NAFLD) is the most common form of liver disease and is characterized by fat accumulation in the liver. Hypercaloric diets generally increase hepatic fat accumulation, whereas hypocaloric diets decrease liver fat content. In addition, there is evidence to suggest that moderate amounts of unsaturated fatty acids seems to be protective for the development of a fatty liver, while consumption of saturated fatty acids (SFA) appears to predispose toward hepatic steatosis. Recent studies highlight a key role for mitochondrial dysfunction in the development and progression of NAFLD. It is proposed that changes in mitochondrial structure and function are key mechanisms by which SFA lead to the development and progression of NAFLD. In this review, it is described how SFA intake is associated with liver steatosis and decreases the efficiency of the respiratory transport chain. This results in the production of reactive oxygen species and damage to nearby structures, eventually leading to inflammation, apoptosis, and scarring of the liver. Furthermore, studies demonstrating that SFA intake affects the composition of mitochondrial membranes are presented, and this process accelerates the progression of NAFLD. It is likely that events are intertwined and reinforce each other, leading to a constant deterioration in health.

## Graphical abstract

Mitochondrial dysfunction is a key mechanism by which saturated fatty acids (SFA) result in the development and progression of non‐alcoholic fatty liver disease (NAFLD). SFA increases liver fat, decreases the efficiency of the respiratory transport chain and results in the production of reactive oxygen species and endoplasmatic reticulum stress. SFA also affect the composition of mitochondrial membranes. These processes lead inflammation, apoptosis, and scarring of the liver.

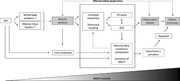

## 1 Introduction

Non‐alcoholic fatty liver disease (NAFLD) is an umbrella term used to describe conditions characterized by excessive accumulation of fat in the liver, the causes of which are not attributable to alcohol consumption. NAFLD is the most common liver disease and occurs in ≈20–40% of all adults in the US,^[^ ^1^ ^]^ in up to ≈70% of adults who are overweight and in >90% of adults who are morbidly obese.^[^ ^2^, ^3^ ^]^ Hepatic steatosis is the first phase of NAFLD and is diagnosed when more than 5% of the liver weight consists out of fat. Although simple fat accumulation in the liver was previously considered benign, it is now clear that hepatic steatosis is an important health problem that can lead to more advanced stages of NAFLD, which include nonalcoholic steatohepatitis (NASH), fibrosis, cirrhosis, and hepatocellular carcinoma. In addition, hepatic steatosis predisposes toward the development of insulin resistance,^[^ ^4^ ^]^ cardiovascular diseases,^[^ ^5^ ^]^ and premature death.^[^ ^6^ ^]^

The amount of fat in the liver is regulated by the delivery of lipids to the liver and the hepatic uptake, synthesis, oxidation, and secretion of lipids and alterations in the equilibrium of one or more of these processes can result in hepatic steatosis.^[^ ^7^ ^]^ To date there are few studies that investigated the contributions of the different pathways to hepatic steatosis in detail.^[^ ^8^ ^]^ An elegant study performed by Donelly et al. included patients that were obese and diagnosed with NAFLD and found that 60% of hepatic fat originated from adipose tissue fatty acids release, while 25% came from de novo lipogenesis, and the remainder from the diet.^[^ ^8^ ^]^ In healthy lean individuals these values may differ though. Lean individuals have decreased adipose tissue mass compared to obese individuals, which results in a decreased flux of adipose tissue derived fatty acids to the liver.^[^ ^9^ ^]^ In addition, lean individuals have a higher insulin sensitivity, which leads to a more pronounced inhibition of adipose tissue lipolysis after a meal. Furthermore, while obese individuals maintain a high rate of de novo lipogenesis throughout the day, the rate of de novo lipogenesis in healthy individuals has been shown to decrease from ≈23% 4 h after eating to ≈5% in the fasted condition.^[^ ^10^ ^]^ Dietary intake, including total energy intake, macronutrient composition of the diet, carbohydrate quality, and fat quality, is also an important determinant of fat deposition in the liver (reviewed in [11 and 12]). In recent years, there has been a growing interest regarding the association between NAFLD and the type of fat in the diet, and studies have linked intake of high amounts of saturated fatty acids (SFA) to the development of hepatic steatosis.^[^ ^13^, ^14^, ^15^, ^16^, ^17^, ^18^ ^]^ This can be due to a reduced insulin‐mediated inhibition of adipose tissue lipolysis^[^ ^17^, ^19^ ^]^ or a decrease in whole body fat oxidation^[^ ^20^, ^21^, ^22^ ^]^), both of which may lead to an increased flux of FA to the liver.^[^ ^23^, ^24^ ^]^ Furthermore, there are indications that SFA induce mitochondrial dysfunction or maladaptation which leads to the development and progression of NAFLD. Indeed, hepatic mitochondria of mice showed ballooned or rounded cristae as well as condensed matrix structures after a HFD.^[^ ^25^ ^]^ Also, NASH has previously found to be associated with loss of mitochondrial cristae and paracrystalline inclusions in humans.^[^ ^9^, ^26^ ^]^ These changes go hand in hand with alterations in respiratory chain activity and ATP homeostasis, increased formation of reactive oxygen species (ROS) and apoptosis. Also changes in the mitochondrial membrane composition have been found in individuals with NAFLD, which has profound effects on mitochondrial function.^[^ ^27^, ^28^ ^]^ The purpose of this review is to assess studies that have investigated the influence of SFA on NAFLD development, and to assess the role of mitochondrial dysfunction herein.

## 2 Framework of the Review

There is a considerable amount of data available on the effect of different types of diets varying in caloric content, macronutrient content and macronutrient composition on the development of NAFLD. There is less data available regarding the specific role of SFA in the development of NAFLD. A literature search was performed between October 2019 and May 2020 using the database “PubMed.” The search strategy consisted of a combination of the following search terms using the Boolean operator “AND” and “OR”: “fatty liver, NAFLD, liver steatosis, diet, dietary fats, fat quality, SFA, mitochondrial dysfunction, mitochondrial membrane, ROS, endoplasmatic reticulum (ER) stress, cardiolipin, ceramides, and apoptosis.” Articles in **Table** **1** were included if studies were human intervention studies that investigated the effect of dietary fat quality on liver fat (SFA versus mono‐unsaturated fatty acids (MUFA) or SFA versus poly‐unsaturated fatty acids (PUFA)). Articles in Table 1 were excluded if the diets of the treatment group (SFA) and the control group differed with respect to caloric content or macronutrient content. Our review will start with an overview of studies showing that diets high in SFA result in the development of liver steatosis. Subsequently, a description will be given on how mitochondrial morphology and function is impaired in patients with NAFLD. Thereafter, we will provide evidence that supports the notion that SFA play a causal role in the development of mitochondrial dysfunction, which will drive the progression of NAFLD. The final paragraph will discuss important gaps in literature and potential topics for future research.

**Table 1:** Human intervention studies comparing the effect of SFA and PUFA on liver steatosis

Subjects	Intervention	Duration	Body weight	Liver fat	Other observations	Reference
39 healthy men and women age: 20–38 y BMI: 18–27 kg m^2^	Randomized control study; overfeeding (+750 kcal per day) SFA (palmitate, 16:0)) or PUFA (linoleate 18:2 n‐6)) muffins. (Muffins were added to the habitual diet).	7 weeks	SFA: 1.6 kg ↑ PUFA: 1.6 kg ↑	SFA ↑ PUFA ↔	SFA: larger increase in visceral adipose tissue compared with PUFA PUFA: increase in lean tissue	[14]
60 healthy men and women Age: 20–55y BMI: 25–32 kg m^2^	Randomized control study; overfeeding (+700 kcal per day) SFA (palmitate, 16:0)) or PUFA (linoleate 18:2 n‐6)) muffins. (Muffins were added to the habitual diet).	8 weeks	SFA: 2.3 kg ↑ PUFA: 2.6 kg ↑	SFA ↑ PUFA ↔	SFA: increased serum ceramides PUFA: reduced serum ceramides. No changes in markers of inflammation, oxidative stress or endothelial function.	[15]
67 men and women with and without T2D Age: 30–65 y BMI: ≈30 kg m^2^	Randomized control study; diet high in SFA (butter) or PUFA (linoleate; ≈6.7 E%) intake. Modest increase in total energy intake (PUFA: +138 kcal per day; SFA: + 225 kcal per day).	10 weeks	SFA: 0.8 kg ↑ PUFA: 0.4 kg↑	SFA ↑ PUFA ↓	Modest increase in inflammatory markers after SFA compared with PUFA. No difference in oxidative stress markers between the diets.	[16]
39 healthy men and women age: 18–65 y BMI: 27–35 kg m^2^	Randomized control study; overfeeding (+1000 kcal per day). SFA or UFA (MUFA and PUFA).	3 weeks	SFA: 1.4 kg ↑ PUFA: 0.9 kg↑	SFA ↑↑ UFA ↑	Increase in insulin resistance (clamp) and circulating ceramides in SFA.	[17]

Only studies are selected in which treatment groups within studies are similar in energy intake and macronutrient content. Abbreviations: y: year; BMI: body mass index; kg: kilogram; m: meter; kcal: kilocalories: SFA: saturated fatty acids; PUFA: poly‐unsaturated fatty acids.

## 3 SFA Lead to the Development of Liver Steatosis in Humans

### 3.1 The Role of Fat Quantity and Quality in Relation to the Development of Liver Steatosis in Humans

Hepatic steatosis in humans can occur within days after initiation of a high‐fat diet. A 2‐week isocaloric high fat diet (56% total energy from fat) in obese females increased liver fat by 35% compared to baseline, while an isocaloric low‐fat diet (16% total energy from fat) decreased liver fat by 20%.^[^ ^29^ ^]^ In a study in which the regular diets of healthy males were supplemented with 800 mL cream for a period of only 3 days, liver fat increased in the absence of significant weight gain.^[^ ^30^ ^]^ It has been shown that even a single energy‐dense high fat meal in the form of sausages rolls (61.5% energy from fat) or in a liquid form induced net lipid accumulation in the liver.^[^ ^31^, ^32^ ^]^ Interestingly though, there are also studies that do not support a role for a high fat diet per se in the development of liver steatosis. For example, a diet that was in energy balance but very high in fat and saturated fat had no effect on hepatic triglyceride levels in overweight individuals,^[^ ^33^ ^]^ whereas diets that were in energy balance but high in MUFA even decreased levels of liver fat in patients with pre‐T2D,^[^ ^34^ ^]^ T2D,^[^ ^35^ ^]^ and NAFLD.^[^ ^36^, ^37^ ^]^ The reason for the inconsistency between studies may in part be explained by the energy content of the diet. Studies using hypercaloric diets show in general an increase in hepatic fat accumulation,^[^ ^30^ ^]^ whereas hypocaloric diets decrease liver content^[^ ^37^ ^]^ (reviewed in [11 and 12]). Apart from the calorie content of the diet, there is also evidence to suggest that the type of fat consumed affects the development of liver steatosis. A number of cross‐sectional and longitudinal studies found a positive link between SFA and liver fat, and an inverse association for PUFA and liver fat. For example, using logistic regression analysis (adjusted for sex and age), associations were assessed between circulating fatty acids and liver fat content in ≈2000 young, Finnish adults. The authors found a robust inverse association between serum PUFA and liver fat whereas a positive association was observed between serum SFA and liver fat.^[^ ^38^ ^]^ Interestingly, serum fatty acid saturation in this study was also predictive of the 10‐year risk for fatty liver even after adjusting for baseline waist circumference, BMI, smoking, physical activity, and alcohol intake.^[^ ^38^ ^]^ We recently found that individuals with NASH had significantly more SFA in their liver triacylglycerols compared to the individuals without NAFLD, whereas the amount of PUFA was progressively lower in accordance with NAFLD severity.^[^ ^39^ ^]^ Also Puri et al. found a threefold increase in absolute levels of total SFA in livers of patients with NAFLD, with no further increase in patients with NASH.^[^ ^40^ ^]^ Interestingly, Sobrecases et al. found that a hypercaloric diet high in SFA (+30% of total energy as fat of which +18% as SFA) increased liver fat more than a hypercaloric diet high in fructose (+35% of energy) (≈86% and ≈16% change in liver fat content respectively).^[^ ^13^ ^]^ This is remarkable, as many studies considered fructose as one of the most detrimental substrates to stimulate de novo lipogenesis and to result in the accumulation of liver fat. In line with this, a very recent study showed that also an isocaloric diet enriched in SFA increased liver fat and postprandial glycaemia, whereas an isocaloric diet high in sugar had no effect on liver fat and induced only minor metabolic changes.^[^ ^18^ ^]^

### 3.2 Controlled Human Diet Intervention Studies

To date, there are to our knowledge only four human diet intervention studies that compared the effect of dietary FA saturation on the development of liver steatosis with the inclusion of an appropriate control group, and without differences in macronutrient content between the treatment groups (Table 1).^[^ ^14^, ^15^, ^16^, ^17^ ^]^ Nevertheless, the results are unequivocal and show a deterioration in liver fat content after the consumption of diet enriched in SFA compared with PUFA. Rosqvist et al.^[^ ^14^ ^]^ performed an elegant double‐blind, randomized, controlled trial in which lean individuals were overfed with muffins high in either palm (SFA) or sunflower oil (PUFA) (Table 1). The muffins accounted for an additional caloric intake of ≈750 kcal per day, and apart from fat quality, they were identical with regard to energy, fat, protein, carbohydrate, cholesterol content, taste, and structure. The authors showed that 7 weeks overfeeding caused similar weight gain in both groups, but while PUFA resulted in an increase in lean body mass, consumption of SFA resulted in increased fat deposition in liver and visceral adipose tissue.^[^ ^14^ ^]^ Later on, the same authors repeated this study protocol in overweight and obese individuals, and found that the effects of SFA and PUFA on liver fat were even more distinct compared to the lean subjects. Overconsumption of SFA resulted in a >50% increase in liver fat as well as increased serum ALT and serum ceramides levels, whereas overfeeding PUFA did not affect hepatic lipid levels and improved the blood lipid profile. If anything, PUFA overfeeding resulted in a slight decrease in liver fat content.^[^ ^15^ ^]^ Bjermo et al.^[^ ^16^ ^]^ assigned obese individuals to a 10‐week isocaloric diet rich in SFA or PUFA, also without altering the carbohydrate, fat, and protein content. Despite the isocaloric diet, there was a modest but similar increase in energy intake in both groups, which resulted in modest increase in body weight. Nevertheless, liver fat was lower after the PUFA diet than after the SFA diet, and this was in spite of a liver fat content of <5% in most participants.^[^ ^16^ ^]^ Also Luukonen et al.^[^ ^17^ ^]^ found in overweight males that 3‐weeks overfeeding SFA (+1000 kcal per day) was more harmful for the liver than unsaturated fatty acids (UFA) and simple sugars, as shown by an increase in liver fat content (SFA: +55%, UFA: +15%, sugar: +33%), and an increase in insulin resistance.^[^ ^17^ ^]^

### 3.3 Mechanisms through Which SFA Increases Liver Fat

**Figure 1:**
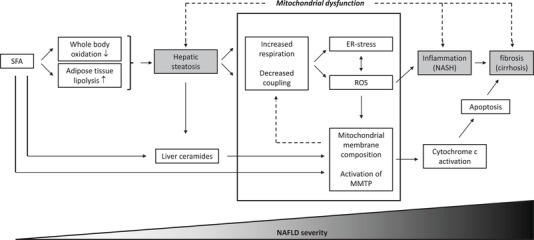
Changes in mitochondrial structure and function are key mechanisms by which SFA lead to the development and progression of NAFLD. Saturated fat intake lead to liver steatosis via decreased whole body oxidation and increased adipose tissue lipolysis. Liver steatosis decreases the efficiency of the respiratory transport chain and results in the production of reactive oxygen species and ER‐stress. This results in damage to nearby structures, eventually leading to inflammation, apoptosis, and scarring of the liver. Furthermore, SFA affects the composition of mitochondrial membranes, and this process accelerates the progression of NAFLD. It is likely that events are intertwined and reinforce each other, leading to a constant deterioration in health.

A study in overweight humans aimed to investigate why a high intake of SFA may lead to hepatic fat storage as opposed to PUFA. It was found that a 3‐week diet enriched in SFA reduced the suppression of lipolysis in adipose tissue as measured during a hyperinsulinemic euglycemic clamp and increased the expression of proteins involved in adipose tissue lipolysis,^[^ ^17^, ^19^ ^]^ compared with a diet enriched in PUFA. It has been hypothesized that this may enhance lipid accumulation in the liver (**Figure** **1**).^[^ ^24^ ^]^ Moreover, SFA may decrease whole body FA oxidation compared to PUFA. A few decades ago, Jones et al. provided young healthy male subjects with a ^13^C‐labeled capsule of stearic, oleic or linoleic acid with breakfast to determine the impact of different FAs on postprandial oxidation rate. Breath samples were analyzed for ^13^CO_2_ abundance over a period of 9 h and showed that SFA were oxidized at a slower rate compared to PUFA.^[^ ^20^ ^]^ In a follow‐up study, indirect calorimetry measurements were performed in young healthy males to investigate the effect of diets varying in saturated/polysunsaturated FA composition on whole body substrate oxidation.^[^ ^21^ ^]^ The authors found that a 7‐day diet high in SFA decreased fat oxidation in the fasted as well as in the post‐prandial condition, and resulted in a decreased diet‐induced thermogenesis when compared to a diet high in unsaturated FA,^[^ ^21^ ^]^ which was in line with their previous results.^[^ ^20^ ^]^ More recently, DeLany et al. fed ^13^C‐labeled fatty acids to men to determine the impact of fatty acid chain‐length and (un)saturation on postprandial oxidation rate.^[^ ^22^ ^]^ After consuming a weight‐maintenance diet for 1 week, subjects were fed different fatty acids labeled with ^13^C in the methyl or carboxyl position. The fatty acids were laurate (12:0), palmitate (16:0), stearate (18:0), oleate (cis 18:1 ln‐9), elaidate (trans 18:1 n‐9), linoleate (18:2, n‐6), and linolenate (18:3, n‐3), and were blended in a hot liquid meal. The authors found, regardless of the place of ^13^C labeling, that SFA were less highly oxidized than unsaturated fatty acids, that oxidation of SFA was inversely related to carbon length, and that there was a nearly perfect positive linear relation between oxidation and the number of double bonds.^[^ ^22^ ^]^ Thus, the results Jones et al.^[^ ^20^, ^21^ ^]^ and DeLany et al.^[^ ^22^ ^]^ suggest that SFA lead to a decreased whole body fat oxidation rate compared to unsaturated FA's, which may lead to an increased flux of dietary FA to the liver (Figure 1).^[^ ^23^ ^]^ In support of this, it has also been shown in hepatocytes that PUFA induce changes that direct FA's away from triacylglycerol storage, and favor FA entry and oxidation into mitochondria.^[^ ^41^ ^]^ Specifically PUFA lead to an upregulation of the expression of genes encoding proteins involved in fatty acid oxidation while simultaneously downregulating genes encoding proteins of lipid synthesis.^[^ ^41^ ^]^ Interestingly though, Rosqvist et al.^[^ ^15^ ^]^ did not find a difference in hepatic fat oxidation between subjects fed a SFA and PUFA (muffin overfeeding, see Table 1), and they also found no difference in fasting or postprandial non‐esterified fatty acids between groups, suggesting that these pathways may not be the primary mediators of the differential effects on liver fat.^[^ ^15^ ^]^ De novo lipogenesis in healthy individuals was also not differentially affected by a 3‐week hypercaloric diet (+1000 kcal) high in SFA compared with a diet high in PUFA.^[^ ^17^ ^]^ More research will be necessary to determine the exact pathways through which SFA intake increases liver fat. Of note, it also remains to be established whether all SFA have similar effects on the development of liver steatosis. About 70% of the fat in milk consist of SFA,^[^ ^42^ ^]^ and yet, intake of full fat milk has been found to be associated with decreased liver fat and positive metabolic health outcomes.^[^ ^43^ ^]^ Possibly, the beneficial effects may be ascribed to a high level of medium‐chain, odd‐chain, and very long‐chain SFA in dairy products.^[^ ^44^ ^]^ In support of this, it has been shown that laurate (C12:0), a medium‐chain fatty acid that is found in coconut milk and breast milk, is the most highly oxidized fatty acid of all fatty acids.^[^ ^22^ ^]^ Thus, studies needs to investigate whether SFA from dairy sources may indeed have different effects on the development of liver steatosis compared with SFA coming from meat.

## 4 Mitochondrial Morphology and Function is Impaired in Patients with NAFLD

With ≈500–4000 mitochondria per cell, hepatocytes are very rich in mitochondria.^[^ ^45^ ^]^ The turnover of mitochondria in liver is relatively high; studies in rats estimated the mitochondrial half‐life in liver tissue on 9.3 days, compared to 12.6 days in testes, 17.5 days in heart, and 24.4 days in brain.^[^ ^46^ ^]^ Mitochondrial dysfunction is regarded as one of the hallmarks of NAFLD progression.^[^ ^47^, ^48^ ^]^ Findings in literature indicate that alterations in respiratory chain activity, ROS production, lipid peroxidation, and changes in mitochondrial membrane composition are present in individuals with hepatic steatosis and drive the progression of NAFLD.

### 4.1 Respiratory Chain Activity and ATP Homeostasis

Mitochondria are highly adaptive and can increase in number and capacity in environments of substrate excess. During fasting, mitochondrial β‐oxidation in rat hepatocytes has been shown to be upregulated tenfold to accommodate for large differences in lipid influx and insulin action.^[^ ^49^ ^]^ In line with this, it has been found that mice and individuals with hepatic steatosis have increased hepatic fat oxidation and increased mitochondrial respiration.^[^ ^47^, ^50^, ^51^, ^52^, ^53^ ^]^ Using ^2^H and ^13^C tracers, Sunny et al. showed that that mitochondrial oxidative metabolism was approximately twofold greater in patients with simple hepatic steatosis compared with individuals with a healthy liver, and they observed a positive correlation between hepatic fat content and both mitochondrial oxidative and anaplerotic fluxes.^[^ ^50^ ^]^ Also a study performed by Koliaki et al. reported a 4.3‐ to fivefold higher maximal respiration in isolated mitochondria of patients with hepatic steatosis compared to mitochondria of lean individuals.^[^ ^51^ ^]^ Interestingly though, these studies do not indicate that mitochondrial function per se is improved in patients with hepatic steatosis. Hepatic steatosis has been linked to increased mitochondrial uncoupling,^[^ ^51^, ^54^ ^]^ suggesting that the increase in respiration may not be bio‐energetically efficient.^[^ ^51^, ^52^, ^55^ ^]^ In support of this, lower hepatic ATP stores were found in overweight mice^[^ ^54^ ^]^ and obese individuals.^[^ ^56^ ^]^ It has previously been suggested that the increased mitochondrial capacity in the liver of individuals with liver steatosis serves to protect against NAFLD progression, but eventually these mechanisms will fail, resulting in a decline in mitochondrial functionality.^[^ ^51^ ^]^ Indeed, the activity of the mitochondrial respiratory chain complexes was found to be decreased in patients with NASH.^[^ ^51^, ^57^ ^]^ Specifically, studies reported a decreased activity of 37% in complex I, 42% in complex II, 30% in complex III, 38% in complex IV, and 58% in complex V, compared with individuals without NAFLD.^[^ ^57^ ^]^

### 4.2 Reactive Oxygen Species Production

**Figure 2:**
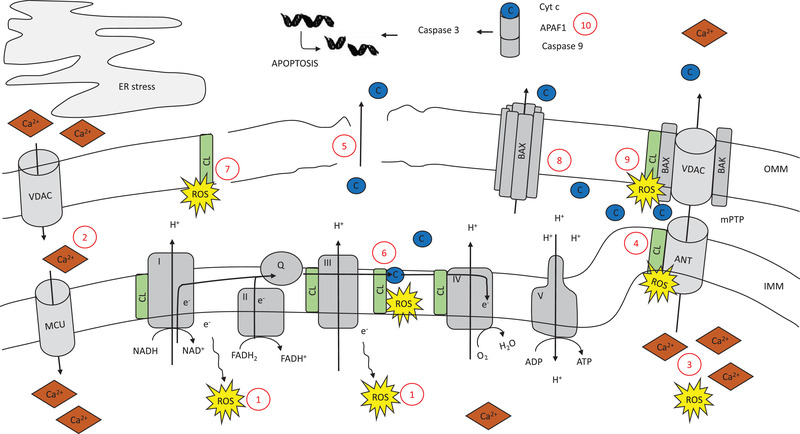
There are several adverse factors within the mitochondria that, alone or together, can result depolarization of the mitochondria, failure of oxidative phosphorylation, ATP depletion, and apoptosis. Oxidative phosphorylation is the process by which electrons from NADH and FADH2 are transferred through a series of electron carriers in order to generate ATP. 1) Electrons that leak out from one of the complexes of the electron transport chain interact with oxygen to form ROS. Influx of Ca^2+^ from the ER into the mitochondria is regulated by VDAC and MCU. 2) In times of ER‐stress, influx of Ca^2+^ is increased. mPTP consists of VDAC and ANT. Brief, reversible mPTP opening constitutes a housekeeping function by releasing the mitochondria from accumulated ROS and Ca^2+^. 3) Increased levels of ROS and Ca^2+^, however, leads to longer mPTP openings, depolarization, and release op apoptotic factors into the cytosol. Cardiolipin is tightly bound to ANT. 4) Interaction of perioxidized cardiolipin with ANT also leads to activation of the mPTP. 5) Increased mPTP opening can lead to an osmotic imbalance that induces the swelling of the mitochondrial matrix and rupture of the OMM. Cardiolipin is tightly bound to several protein complexes on the IMM, including cytochrome c. 6) Changes in the composition or content of cardiolipin as well as peroxidation of cardiolipin lead to detachment of cytochrome c from cardiolipin, and translocation of cardiolipin to the OMM. 7) Cardiolipin translocation destabilizes the OMM lipid composition which favors the formation of pores, and leads to a disturbed equilibrium of the mitochondria. Cardiolipin translocation to the OMM also results in the recruitment of BAX. 8) BAX assembles into large oligomers and induces pores in the OMM. 9) BAX also works in conjunction with BAK to facilitate the opening of mPTP. In the cytoplasm, cycochrome c binds to the cytosolic protein APAF1 to promote the formation of an “apoptosome,” which leads to activation of caspase‐9 and caspase 3. 10) This results in DNA fragmentation and chromatin condensation.

Mitochondria not only produce energy, but are also a major cause of the production of ROS within a cell. ROS are formed when electrons leak out from one of the complexes from the electron transport chain. The electrons can interact with oxygen to form superoxides, and damage mitochondria by peroxidizing phospholipid acyl chains, mitochondrial DNA, and enzymes of the respiratory transport chain (**Figure** **2**). In healthy cells, redox molecules within the matrix of the mitochondria will neutralize most of the ROS by converting them into water. However, chronic activation of mitochondrial function in the setting of lipid overload may lead to excessive leakage of electrons from complex I and III of the electron transport chain, which predisposes the liver to high amounts of oxidative stress.^[^ ^47^, ^53^, ^55^ ^]^ The mitochondrial permeability transition pore (mPTP), is a pore through the mitochondrial membranes that consist of the protein adenine nucleotide translocator (ANT) on the inner mitochondrial membrane (IMM), the voltage‐dependent anion channel (VDAC) on the outer mitochondrial membrane (OMM).^[^ ^58^, ^59^ ^]^ Induction of mPTP leads to an abrupt and brief opening in the permeability of the IMM, and serves to release the mitochondria from potentially toxic levels of ROS. However, increased ROS levels result in a prolonged opening of the mPTP and this will lead to depolarization of mitochondria, failure of oxidative phosphorylation, ATP depletion and the release of pro‐apoptotic factors.^[^ ^60^ ^]^ Increased mPTP opening can also lead to an osmotic imbalance that induces swelling of the mitochondrial matrix and results in rupture of the OMM (Figure 2)^[^ ^58^, ^60^, ^61^ ^]^ These processes will result in the destruction of mitochondria or even the cell itself, and will drive the progression of NAFLD. Increased levels of ROS and lipid peroxidation has been shown to be increased in individuals with hepatic steatosis, and even higher values were found for individuals with NASH.^[^ ^9^ ^]^ Levels of 8‐hydroxydeoxyguanosine (8‐OHdG) and 4‐hydroxy‐2'‐nonenal (HNE) are used as markers of lipid peroxidation and oxidative DNA damage, and were measured in individuals with and without NAFLD. There was no 8‐OHdG expression observed in healthy liver and 8‐OHdG expression was only found in two out of the 23 individuals with a fatty liver. In contrast, most individuals with NASH (11 of 17 cases) showed nuclear expression of 8‐OHdG in areas of active inflammation, and the 8‐OHdG index significantly correlated with the grade of necro‐inflammation.^[^ ^62^ ^]^ There were no HNE adducts observed in healthy livers. However, they were frequently detected in individuals with advanced NAFLD and they correlated significantly with the grade of necro‐inflammation as well as the stage of fibrosis. Also another study showed increased hepatic mitochondrial uncoupling and leaking activity in patients with NASH, and the increase in 8‐OHdG was paralleled by an increase in systemic inflammation.^[^ ^51^ ^]^ These findings suggest that ROS production followed by lipid peroxidation and apoptosis are processes that are present in individuals with hepatic steatosis and drive the progression of NAFLD.

### 4.3 Mitochondrial Membrane Composition

The composition of the IMM and OMM are very different from each other. The composition of the OMM is relatively similar to the composition of the cell membrane and contains high concentrations of phosphatidylcholine (PC) phosphatidylethanolamine (PE) and phosphatidylinositol (PI). The IMM also contains large quantities of PC and PE, but only low quantities of PI. Additionally, cardiolipin is only present in minimal amounts on the OMM,^[^ ^63^ ^]^ whilst it constitutes ≈20% of the total mitochondrial phospholipid content in the IMM. Cardiolipin is an important phospholipid that is critical in many reactions and processes related to mitochondrial function and dynamics (reviewed in [64]). Cardiolipin interacts with and stabilizes mitochondrial enzymes on the IMM, including enzyme complexes of the electron transport chain and ATP production.^[^ ^64^, ^65^, ^66^, ^67^ ^]^ It is also important for maintaining inner membrane fluidity and osmotic stability^[^ ^64^, ^68^ ^]^ and it plays a role in the assembly of respiratory enzyme supercomplexes.^[^ ^64^, ^69^ ^]^ Several studies report increased hepatic cardiolipin levels upon a HFD in rats and humans.^[^ ^39^, ^70^, ^71^ ^]^ Mitochondrial cardiolipin content in livers of HFD mice started to increase in the first weeks after initiation of the diet. They reached maximal values after 12 weeks and returned toward control values at 20 weeks of the diet.^[^ ^71^ ^]^ The mitochondrial cardiolipin content was positively correlated to in vitro measures of mitochondrial membrane potential and respiration, which suggests that the increase in cardiolipin levels might be an attempt to preserve mitochondrial function in response to the excessive energy substrate availability and/or hepatic insulin resistance.^[^ ^39^, ^71^ ^]^ Interestingly, apart from an increase in cardiolipin levels, HFD also affect the structural uniformity and molecular symmetry among cardiolipin species, which has been linked to the development of mitochondrial dysfunction.^[^ ^72^, ^73^ ^]^ The cardiolipin molecule consists of two *sn*‐glycerol‐3‐phosphate moieties linked by a glycerol group, and four acyl groups are attached to the glycerol‐3‐phosphates. The most abundant cardiolipin species in liver mitochondria, representing ≈55 % of total liver cardiolipin species, is tetralinoleoyl–cardiolipin, in which all four acyl groups are derived from linoleate (C18:2). Tetralinoleoyl–cardiolipin deficiency has been shown to be a specific marker of Barth syndrome, which is a childhood onset of dilated cardiomyopathy and neutropenia associated with mitochondrial respiratory chain dysfunction,^[^ ^74^ ^]^ and reductions in tetralinoleoyl–cardiolipin levels have also been linked to mitochondrial dysfunction in mice and individuals with cardiac failure^[^ ^75^, ^76^ ^]^ We observed cardiolipin remodeling in livers of individuals with NAFLD.^[^ ^39^ ^]^ Specifically, the percentage of linoleate in the cardiolipin pool was lower in individuals with NASH, while the percentage of monounsaturated fatty acids was found to be increased.^[^ ^39^ ^]^

The precise mechanisms by which changes in cardiolipin content and structure affect mitochondrial function are complex and diverse, and are still topic of investigation. Nonetheless, it has been shown that cardiolipin that is rich in linoleate binds with high affinity to a multitude of proteins and enzymes within the IMM, including key respiratory chain enzymes such as cytochrome c.^[^ ^64^, ^77^ ^]^ Cytochrome c organizes the transfer of electrons from complex III to complex IV, and is a major player in the regulation of cell apoptosis. In healthy conditions, cytochrome c is tightly bound to cardiolipin. However, when the fatty acyl chain composition of cardiolipin changes, cytochrome c detaches from cardiolipin and cardiolipin relocates to the OMM. The presence of cardiolipin in the OMM destabilizes the lipid composition of the membrane, and this favors the formation of pores,^[^ ^59^ ^]^ leading to a disturbed equilibrium in the mitochondria (Figure 2). Cardiolipin translocation to the OMM also promotes conformational changes in the proteins BCL2‐associated X (Bax) and BCL2‐antagonist/killer (Bak). Upon activation, BAK and BAX are believed to facilitate the opening of mPTP, resulting in the release of cytochrome c into the cytosotol^[^ ^58^, ^59^ ^]^ (Figure 2). Recruitment of BAX has also been shown to result in the formation of pores in the OMM, independent of BAK and mPTP, through formation of large oligomers (Figure 2).^[^ ^59^ ^]^ Once cytochrome c is released in the cytosol, it binds to the protein APAF1 to promote the formation of an “apoptosome,” a molecular platform for the activation of caspase‐9. Subsequently, caspase‐9 catalyzes the activation of caspase‐3, which leads to DNA fragmentation and chromatin condensation. Caspase 3 may also help to promote further cytochrome c release from mitochondria, and amplifies as such the initial death signal (Figure 2).^[^ ^59^ ^]^

Another important characteristic of cardiolipin molecules is that they are very susceptible to ROS attacks.^[^ ^66^, ^78^, ^79^ ^]^ This is due to their location in the IMM close to complex I and III, but also because of the presence of double bonds within their structure. Given the fact that high fat feeding increases total hepatic cardiolipin levels^[^ ^39^, ^70^, ^71^ ^]^ and also increases the percentage of long‐chain monounsaturated and polyunsaturated acyl chains in cardiolipin,^[^ ^39^, ^73^, ^76^ ^]^ the risk on ROS attacks increases even more. Also peroxidation of cardiolipin may change its biophysical properties and may lead to a redistribution from the IMM to the OMM, leading to a further decline in mitochondrial metabolism, mobilization of cytochrome c, and apoptosis,^[^ ^77^, ^80^ ^]^ as previously explained (Figure 2).

### 4.4 ER Stress

In the past few decades, electron microscopy studies discovered close proximity between rough ER and mitochondria. It has been found that ≈80% of the mitochondria are in contact with the RER via mitochondria‐associated membranes (MAMs) and these processes are important in order to exchange metabolites and calcium, and regulate cellular homeostasis and signaling. VDAC regulates the transport of Ca^2+^ at the MOM, whereas the mitochondrial Ca^2+^ uniporter (MCU) mediates transport across the IMM into the matrix.^[^ ^81^ ^]^ MAM integrity however has shown to be altered in palmitate‐treated HuH7 cells, and is also affected in the liver of ob/ob and HFD‐fed mice.^[^ ^82^ ^]^ The implications of this are not widely investigated, but there is evidence that this leads to increased influx of Ca^2+^ into the mitochondria, which promotes the production of NADH by enzymes in the TCA cycle, and increases ATP synthesis and ROS production. In addition, increased levels of Ca^2+^ in the mitochondrial matrix induce mPTP opening and result in apoptotic processes in the long term.^[^ ^83^, ^84^ ^]^ (Figure 2).

## 5 Dietary SFA and Mitochondrial Dysfunction in NAFLD

### 5.1 Reactive Oxygen Species Production

Although the role of SFA in relation to mitochondrial function is significantly less studied compared to the role of UFA, there is clear evidence that SFA impair mitochondrial function and drive the progression of NAFLD (Figure 1). A series of experiments in H4IIIC3 liver cells and HepG2 cells aimed to investigate the role of the SFA palmitate in relation to oxidative damage and cell death, and found that palmitate initially enhanced mitochondrial metabolism. In line with this, it is interesting to note that also in a human study, infusion with palm oil resulted in increased hepatic ATP levels in healthy lean individuals.^[^ ^32^ ^]^ These findings are consistent with the finding that respiratory chain activity is initially increased in individuals with hepatic steatosis, as mentioned previously in this review.^[^ ^50^, ^51^ ^]^ The in vitro studies also found that ROS accumulation occurred downstream of altered mitochondrial oxidative metabolism and this preceded the initiation of apoptosis, as shown by an increased caspase 3 activity, and reduced cell viability.^[^ ^85^, ^86^, ^87^ ^]^ Cells treated with the same concentration of oleate did not exhibit markers of oxidative stress or apoptosis.^[^ ^85^ ^]^ Interestingly, treatment with metformin and a low concentration of rotenone ‐a known inhibitor of mitochondrial respiratory chain complex Ι‐ protected against palmitate‐induced hepatic cell death through a moderate inhibition of mitochondrial respiration, as indicated by reduced basal and maximal mitochondrial respiration and proton leak. Furthermore, mitochondrial membrane potential was preserved, which reduced the production of ROS, and increased the expression of superoxide dismutase 2 expression, which is indicative of ROS scavenging.^[^ ^86^ ^]^ These experiments show a critical role for a dysregulated mitochondrial function in mediating palmitate liptoxicity of liver cells.

### 5.2 Changes in Mitochondrial Membrane Composition

There are a number of studies showing that dietary FA's influence the composition of the mitochondrial membranes in liver.^[^ ^77^ ^]^ Supplementation of coconut oil to a diet resulted in a rapid increase in the saturated/unsaturated ratio in hepatic mitochondria of chicken, and the rise in C12:0 and C14:0 reflected the high content of these FA in the diet.^[^ ^88^ ^]^ Rats fed a diet containing 20% rapeseed oil, showed noticeable differences in hepatic mitochondrial membrane lipid composition compared to rats fed a standard diet, as shown by a decrease in the saturated to unsaturated molar ratio, and changed the relative proportions of phospholipid classes.^[^ ^89^ ^]^ Studies also found that dietary fat quality can modulate tetralinoleoyl–cardiolipin levels in heart and in liver of mice and rats.^[^ ^73^, ^77^, ^90^, ^91^ ^]^ Rats were fed a high‐carbohydrate diet or a high‐fat diet supplemented with high‐linoleate safflower oil or lard until death. The high‐linoleate safflower oil significantly increased linoleic acid content in the left ventricle of the heart compared with the control diet, whereas the lard diet elicited a decrease in linoleic acid content. Interestingly, tetralinoleoyl–cardiolipin levels in this study correlated positively with age of mortality, indicating that lower tetralinoleoyl–cardiolipin levels were associated with decreased survival.^[^ ^92^ ^]^ In a different study, rats were fed a corn‐oil based diet, which is high in linoleic acid, or a sardine‐oil based diet, which is low in linoleic acid. Hepatic tetralinoleoyl–cardiolipin levels were drastically reduced in rats fed the sardine oil based diet, and there was a diminished mitochondrial respiratory function.^[^ ^91^ ^]^ In mice, it was also shown that intake of a HFD high in lard resulted in remodeling of cardiolipin acyl chain composition in liver and this was accompanied by diminished complex I to III respiratory enzyme activity by 3.5‐fold.^[^ ^73^ ^]^ qRT‐PCR analyses in this study demonstrated an upregulation of liver mRNA levels of tafazzin, a well‐known acyltansferase, which contributes to cardiolipin remodeling. Apart from animal studies, also detailed cells studies found that palmitate affected the synthesis of cardiolipin, and this was shown to correlate with the release of cytochrome c from the mitochondria.^[^ ^93^ ^]^ Although more detailed mechanistic studies are needed, these studies support a role for dietary SFA in the development of mitochondrial dysfunction via changes in cardiolipin content and composition (Figure 1).

### 5.3 Ceramides

Ceramides are another major player that link SFA to NAFLD progression via mitochondrial dysfunction (Figure 1). Ceramides are members of the sphingolipid family and are synthesized de novo from SFA.^[^ ^94^ ^]^ SFA treatment increased ceramide synthesis in primary rat hepatocytes,^[^ ^95^ ^]^ and a 3‐week HFD rich in SFA led to excessive sphingomyelin and ceramide accumulation in the liver and hepatic nuclei in rats.^[^ ^96^ ^]^ Rats infused with lard oil in showed a 60% increase in hepatic ceramide content, whereas this did not occur in rats infused with soy oil.^[^ ^97^ ^]^ In the overfeeding study of Rosqvist et al.^[^ ^15^ ^]^ and Luukonen et al.,^[^ ^17^ ^]^ mentioned earlier (Table 1), it was found that the SFA and PUFA diets had markedly different effects on serum ceramides. Overall, SFA increased, whereas PUFA decreased serum ceramide levels, and these changes were already evident at 4 weeks into the intervention. Changes in total liver fat were directly associated with changes in all ceramide species.^[^ ^15^ ^]^ In agreement with this, it was found that individuals with NASH had 33% higher hepatic total ceramide levels compared to individuals with hepatic steatosis, and 50% more than individuals without steatosis.^[^ ^98^ ^]^ Interestingly, hepatic ceramide levels in humans correlated with measures of hepatic oxidative stress and inflammation,^[^ ^98^ ^]^ which included high levels of liver thiobarbituric acid‐reactive substances and increased phosphorylation of JNK. Studies, investigating the link between ceramide accumulation and NAFLD progression reported that accumulation of ceramides induce hepatocyte death^[^ ^61^, ^94^ ^]^ . This cell death was mediated by ER stress^[^ ^99^ ^]^ and by mPTP in an acyl chain‐length, concentration, and time‐dependent manner, leading to mitochondrial failure.^[^ ^61^ ^]^ In support of this, it was shown that inhibition of mPTP prevented ceramide‐induced hepatocyte necrosis.^[^ ^61^ ^]^ Also in the beta‐cells of the pancreas it has been demonstrated that treatment with palmitate resulted in the activation of the apoptotic mitochondrial pathway via formation of ceramides.^[^ ^100^ ^]^ Interestingly though, there are also studies that suggest that ceramides are not a prerequisite to induce SFA‐induced cellular dysfunction. SFA exposure to H4IIIE liver cells resulted in increased ceramide levels, ER stress, and apoptosis, but while inhibition of de novo ceramide synthesis decreased intracellular ceramide levels, it did not reduce ER stress or apoptosis.^[^ ^99^ ^]^ This latter finding suggests that also other pathways may be important in SFA‐induced cell death, and imply that ER stress may occur independent of ceramide levels.

## 6 The Role of Unsaturated Fatty Acids in Relation to Mitochondrial Function and NAFLD

Many studies suggest that MUFA and PUFA intake exert beneficial effects on NAFLD, and this is at least partly via improvements in mitochondrial function. Several human intervention studies found that MUFA decreased liver fat,^[^ ^101^ ^]^ whereas controlled cell studies found that MUFA induced hepatic steatosis but did not initiate apoptosis.^[^ ^102^ ^]^ In a double‐blind, randomized, placebo‐controlled trial, in which patients with NASH received 6‐month treatment with n‐3 PUFA, it was found that proteomic markers of cell matrix, lipid metabolism, ER stress, and cellular respiratory pathways were modulated after the treatment period.^[^ ^103^ ^]^ The alterations reflected functional changes highly suggestive of decreased cellular lipotoxicity potential, reduced ER proteasome degradation of proteins and induction of chaperones, and a shift in cell energy homeostasis toward mitochondrial beta‐oxidation.^[^ ^103^ ^]^ Studies in animals and cells support the notion that omega‐3 PUFA improve mitochondrial morphology and exert beneficial effects on the recovery of mitochondrial function.^[^ ^104^, ^105^ ^]^ In an in vitro steatotic hepatocyte model using HepG2 cells, treatment with omega‐3 PUFA increased the expression of Mitofusion 2, a protein that helps determine the shape and morphology of mitochondria. Furthermore, there was an increase in the length of mitochondrial tubules, increased levels of ATP, and decreased production of ROS.^[^ ^104^ ^]^ Several human intervention studies found that also n‐6 PUFA intake did not change or even reduced hepatic fat accumulation.^[^ ^14^, ^15^, ^16^ ^]^ Importantly though, while these studies suggest a positive role for the intake of n‐6 PUFA, attention should be paid to the fatty acid n‐3/n‐6 ratio of the diet, since a decrease in the n‐3/n‐6 ratio has been found to favor a pro‐inflammatory state and leads to adverse metabolic health outcomes,^[^ ^106^, ^107^ ^]^ including hepatic steatosis.^[^ ^108^ ^]^ In line with this, it was found that mice fed a HFD diet with a high ratio n‐3/n‐6 PUFA improved mitochondrial function compared with mice that received a high level of SFA or low levels of n‐3/n‐6 PUFA. The mice that were fed a high ratio n‐3/n‐6 PUFA also showed up‐regulated mitochondrial electron transport chain and tricarboxylic acid cycle pathways, increased mitochondrial complexes activity, reduced fumaric acid levels, and decreased levels of oxidative stress.^[^ ^105^ ^]^ This topic regarding the n‐3/n‐6 ratio is, however, complex and outside the scope of this review, and we refer to other reviews for more detailed information.^[^ ^106^, ^107^, ^108^ ^]^

## 7 Conclusions

In this review, we present evidence to suggest that SFA lead to hepatic lipid accumulation and lipotoxic mechanisms, thereby affecting hepatic mitochondrial structure and function. We propose that this may be a key mechanism by which SFA lead to the development and progression of advanced NAFLD. The road to advanced stages of NAFLD, including NASH and cirrhosis is complex, and the role of SFA in relation to mitochondrial function is significantly less studied compared to the role of unsaturated FA. Nevertheless, there is accumulating evidence to suggest that SFAs play a key role in the development of hepatic steatosis as well as the progression of NAFLD via changes in the regulation of mitochondrial function. Multiple studies in mice and humans showed that hepatic steatosis initially leads to increased mitochondrial respiration. This mitochondrial respiration, however, is not bio‐energetically efficient, as studies found increased uncoupling, increased leakage of electrons and decreased hepatic ATP levels in individuals with hepatic steatosis. This result in the generation of reactive oxygen species and lipid peroxidation, which cause damage to the respirator transport chain and induce apoptosis. Eventually, a vicious cycle ensues in which more ROS production results in even more severe apoptosis. Apart from inducing ROS production, SFA also alter the composition of the mitochondrial membranes. Cardiolipin is a component of the IMM that is relatively well investigated, and SFA have been shown to affect the content and structure of cardiolipin. Also these processes have been linked to increased ROS accumulation, cytochrome c release and cell death.

## 8 Future Directions

It is clear that more research is needed to determine how SFA can modulate mitochondrial respiration and leakage of electrons and which additional pathways may play a role in the initiation of apoptotic processes. Apart from this, the field is also in need of more human nutritional (isocaloric) intervention studies. Three of the four studies^[^ ^14^, ^15^, ^17^ ^]^ described in Table 1 are overfeeding studies. Study four, performed by Bjermo et al.,^[^ ^16^ ^]^ intended to provide an isocaloric amount of energy. However, also in this study the energy intake increased slightly during the intervention period (PUFA: +138 kcal per day; SFA: + 225 kcal per day), which resulted in a mild increase in body weight in all participants. Interestingly, while the present article was under review, a study was published which showed that humans given an isocaloric diet high in SFA also increased their liver fat and postprandial glycaemia, whereas a isocaloric diet high in sugar had no effect on liver fat and induced only minor metabolic changes.^[^ ^18^ ^]^ Studies are now needed to compare the effect of an isocaloric diet high in SFA with an isocaloric diet high in PUFA on liver fat, and with a stronger focus toward underlying mechanisms. Of note, studies should also look into the possible differential effects of SFA originating from dairy sources as opposed to SFA coming from meat. Consumption of dairy products is linked to positive metabolic health outcomes and decreased liver fat, and laurate, a medium chain SFA that is present in coconut milk and breast milk, is one of the most highly oxidized fatty acids. This suggests that not all SFA are equally detrimental. Unfortunately, liver research in humans is very challenging. This is due to the fact that it is not ethical to take (repeated) liver biopsies in healthy individuals, but also, interpretation and comparison of results can be difficult as diets between and within studies may not only differ in fat quality, but also in total energy intake, macronutrient content, macronutrient composition/quality and duration.^[^ ^12^, ^101^ ^]^ Furthermore, hypercaloric or hypocaloric diets that lead to weight gain or weight loss make it impossible to disentangle the effect of fat quality per se from effects that are due to changes in whole‐body adiposity. It is therefore important that future studies control for these factors and replace PUFA by SFA in a highly standardized manner. Advancement in our understanding on how and which SFA drive hepatic steatosis and NAFLD progression, and in which quantities, may help to refine our current nutritional dietary guidelines and may lead to the development of new therapeutic approaches that are targeted to prevent and treat NAFLD.

## Conflict of Interest

The authors declare no conflict of interest.

## Author Contributions

R.C.R.M. wrote the manuscript. E.E.B. reviewed the manuscript. Both authors have read and approved the final version of the manuscript.
